# Thermodynamic Inhibition of Carbon Dioxide Hydrate with Magnesium Chloride and Methanol: Comparative Phase Equilibrium and PXRD Study

**DOI:** 10.3390/ijms27041792

**Published:** 2026-02-13

**Authors:** Anton Semenov, Rais Mendgaziev, Andrey Stoporev, Timur Tulegenov, Daniil Lednev, Murtazali Yarakhmedov, Vladimir Istomin, Daria Sergeeva, Rawil Fakhrullin

**Affiliations:** 1 Department of Physical and Colloid Chemistry, Gubkin University, 65, Leninsky Prospekt, Building 1, 119991 Moscow, Russiastoporev.andrey89@gmail.com (A.S.); darina2010@mail.ru (D.S.); 2 Moscow Center for Advanced Studies, Kulakova Str. 20, 123592 Moscow, Russia; 3 Center for Petroleum Science and Engineering, Skolkovo Institute of Science and Technology (Skoltech), Bolshoy Boulevard, 30, Building 1, 121205 Moscow, Russia; 4 Institute of Fundamental Medicine and Biology, Kazan Federal University, Kreml uramı 18, Kazan 420008, Republic of Tatarstan, Russia

**Keywords:** gas hydrates, carbon dioxide, magnesium chloride, methanol, phase equilibrium, thermodynamic hydrate inhibitor

## Abstract

Clathrate hydrates of carbon dioxide represent a subject of considerable interest in both fundamental science and the development of promising technologies. The phase behavior of CO_2_ hydrate in the presence of concentrated aqueous solutions remains poorly understood. In this study, we conducted a comprehensive investigation into the impact of magnesium chloride (0–24 mass%) and methanol (0–40 mass%) on the thermodynamic stability of CO_2_ hydrate. New experimental data on the three-phase gas–aqueous solution–gas hydrate equilibrium in the temperature range 243–283 K and pressure range 1–4.5 MPa were obtained. A correlation is proposed for the precise representation of equilibrium pressure–temperature lines. A comparison of the anti-hydrate effect, as indicated by the parameter ∆*T*_h_, of these substances demonstrated that ionic MgCl_2_ exhibits a stronger thermodynamic inhibitory effect on CO_2_ hydrate formation than nonionic MeOH. The results of measuring the melting point of ice at 0.1 MPa for aqueous solutions of MgCl_2_ and MeOH confirmed the thermodynamic consistency of the hydrate equilibrium data. A detailed comparison of the anti-hydrate effect of MgCl_2_ and MeOH in a wide concentration range was performed on hydrates of different gases (CO_2_ and CH_4_). The phase composition of CO_2_ hydrate samples obtained from water and aqueous solutions of MgCl_2_ and MeOH was examined using powder X-ray diffraction (PXRD) at 133 K. The PXRD results indicate the formation of sI CO_2_ hydrate with a cell parameter of 11.86 ± 0.04 Å in all cases.

## 1. Introduction

Gas clathrate hydrates are ice-like inclusion compounds that form when water interacts with small guest molecules (e.g., carbon dioxide, hydrogen sulfide, C_1_–C_4_ hydrocarbons, nitrogen, etc.) under specific temperature and pressure conditions, depending on the system’s composition [1]. The water molecules are interconnected by hydrogen bonds, thereby forming a framework of empty cavities that are filled by guest molecules. These guest molecules interact with water through van der Waals forces. A hydrate structure with unfilled cavities is thermodynamically unstable. Filling the cavities with guest molecules stabilizes the hydrate structure.

Gas hydrates are an interesting subject from both fundamental and applied science perspectives. Presently, hydrate technologies are under development for a variety of applications, including natural gas production [2,3], natural gas storage and transportation [4,5], gas mixture separation [6,7], greenhouse gas sequestration [8,9], seawater desalination [10,11], and others. Currently, particular attention is being directed toward the study of the phase behavior of carbon dioxide hydrates in the context of developing these technologies. Single and mixed carbon dioxide hydrates are promising phase change materials with high specific melting enthalpy [12,13]. In order to utilize the processes of carbon dioxide hydrate formation and decomposition in various technologies, reliable experimental data on phase behavior in different systems are required.

Gas hydrate formation potentially occurs during the processes of oil and gas production and transportation. This phenomenon arises due to the presence of suitable thermobaric conditions and the existence of free water and natural gas components within the flow [14]. These particles tend to agglomerate, forming hydrate plugs that can impede processing pipelines and equipment. Consequently, gas hydrate formation poses a substantial challenge to the oil and gas industry. This challenge is addressed via chemical reagents, such as gas hydrate inhibitors. The predominant class of reagents employed in industry is represented by thermodynamic hydrate inhibitors (THIs). THIs decrease the thermodynamic activity of water in solution when added, causing a shift in the three-phase equilibrium lines (gas–water solution–gas hydrate, or V–L_w_–H) to lower temperatures. In order to apply THIs practically, it is necessary to have reliable experimental data on gas hydrate phase equilibria over a wide range of concentrations.

The thermodynamics of carbon dioxide hydrate formation have been studied in experimental works [15,16,17,18,19,20,21,22,23]. Literature data indicate that thermodynamic inhibitors of carbon dioxide hydrate formation include a wide range of compounds, such as amino acids (e.g., glycine, alanine, valine, proline, serine, arginine, and lysine) [24,25,26,27,28], ionic liquids with various structures [29,30,31,32,33,34,35,36,37,38], lower alcohols and other oxygen-containing compounds (e.g., methanol [19,39,40,41,42], ethanol [43], ethylene glycol [19,39,40,41,42,44], glycerol [39], 1-propanol [40], 2-propanol [40,45], 2-butanol [46], D-sorbitol [47], dimethyl sulfoxide [22,48], and 1,4-cyclohexanedione [49]), salts (e.g., sodium chloride [40,43,44,45,50], potassium chloride [42,50], calcium chloride [41,50,51], and magnesium chloride [19,50,51]), and nitrogen-containing compounds (e.g., N-methyldiethanolamine [52], 2-pyrrolidone [53], and 1,2,4-triazole [54]). A review indicates that the thermodynamics of carbon dioxide hydrate formation have been predominantly studied in dilute solutions within a limited concentration range. In this study, we investigated the phase behavior of CO_2_ hydrate over a wide concentration range by using two thermodynamic inhibitors of different nature: nonionic methanol and ionic magnesium chloride. This comparative analysis examines the influence of these compounds on the thermodynamics of CO_2_ hydrate formation. These findings contribute to the advancement of knowledge in the field of physical chemistry of gas hydrates. These results are pertinent to the potential application of magnesium chloride (the mineral bischofite) as a more effective, natural thermodynamic inhibitor of gas hydrates compared with the ones employed in industry (methanol and ethylene glycol).

## 2. Results

**Figure 1:**
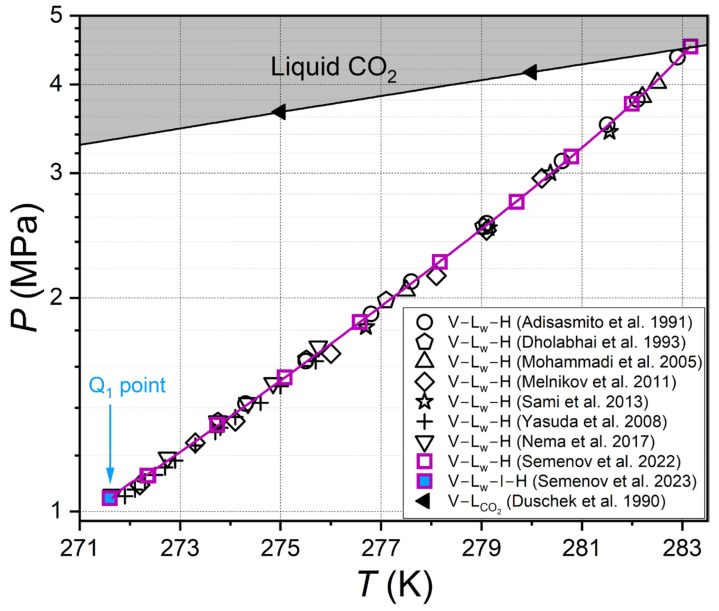
Comparison of V–L_w_–H equilibrium data for the CO_2_–H_2_O reference system [22] with literature findings [15,16,17,18,19,20,21]. Symbols—experimental values; purple line—approximation based on experimental data [22] using Equation (1). The figure also shows the result of the direct measurement of the quadruple point Q_1_ (V–L_w_–I–H equilibrium) [55] and the vapor–liquid equilibrium curve of carbon dioxide [56].

Prior to the investigation of the phase behavior of carbon dioxide hydrate in aqueous solutions of methanol (MeOH) and MgCl_2_, a comparison of the hydrate equilibrium conditions for the CO_2_–H_2_O reference system was made. The results are displayed in Figure 1, in which the measured data from our previous work [22] are compared with literature findings [15,16,17,18,19,20,21]. As demonstrated, our V–L_w_–H equilibrium data are in strong agreement with those reported in the extant literature.

### 2.1. Carbon Dioxide Hydrate Equilibrium Data for Methanol and Magnesium Chloride Aqueous Solutions

**Figure 2:**
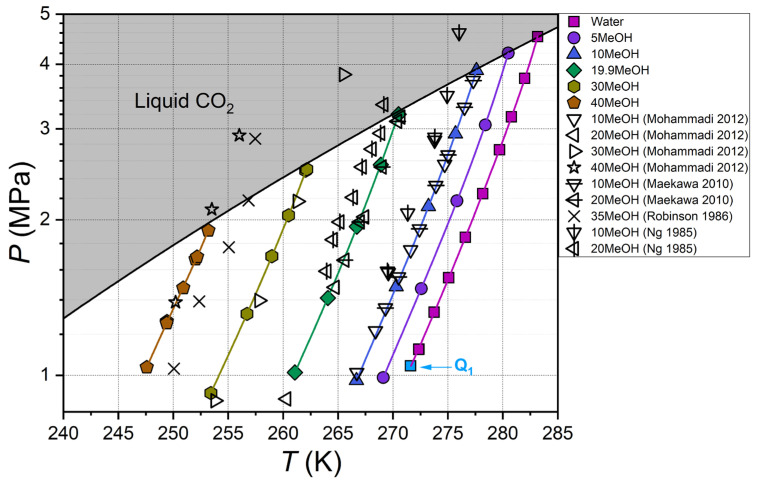
Measured carbon dioxide hydrate equilibrium conditions for aqueous methanol solutions. Color symbols represent data of this work and quadruple point Q_1_ for CO_2_–H_2_O system from [55], black-edge symbols show literature data [39,59,60,61], color lines are approximations based on data from this work (Equation (1), Figure S1 and Table S3), and black line shows vapor–liquid equilibrium for carbon dioxide [56]; legend shows methanol concentration in aqueous solutions in mass%.

**Figure 3:**
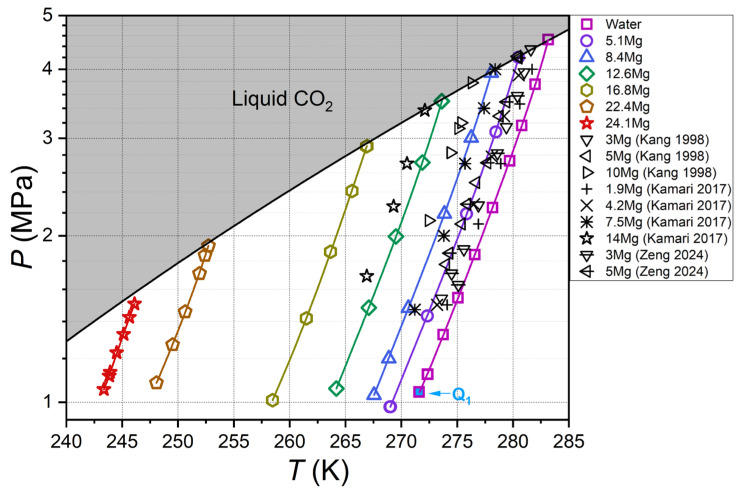
Measured carbon dioxide hydrate equilibrium conditions for aqueous MgCl_2_ solutions. Color symbols represent data of this work and quadruple point Q_1_ for CO_2_–H_2_O system from [55], black-edge symbols show literature data [64,65,66], color lines are approximations based on data from this work (Equation (1), Figure S2 and Table S4), and black solid line shows vapor–liquid equilibrium for carbon dioxide [56]; legend shows MgCl_2_ concentration in aqueous solutions in mass%.

Tables S1 and S2 present the numerical data on the measured equilibrium temperatures and dissociation pressures of CO_2_ hydrate. The results are presented graphically in Figure 2 and Figure 3, in comparison with literature data. Equation (1) was utilized to approximate the temperature–pressure dependence on the V–L_w_–H equilibrium line for each MeOH and MgCl_2_ concentration.

(1) $$T=\frac{a_{1}P^{2}+a_{2}P+a_{3}}{P+b_{1}},$$

In this equation, *T* denotes the equilibrium temperature, *P* symbolizes the equilibrium pressure, and *a*_1_–*a*_3_ and *b*_1_ represent coefficients. This equation accurately describes hydrate equilibrium curves in various systems, including those containing THIs [57,58]. The approximation results for aqueous MeOH solutions are shown in Table S3 and Figure S1. The results for aqueous MgCl_2_ solutions are documented in Table S4 and Figure S2. The determination coefficient, *R*^2^, for MeOH solutions ranges from 0.9997 to 1.0000, and the average absolute deviation (AAD) of the fitted equilibrium temperatures from the experimental ones is between 0.004 and 0.023 K. For MgCl_2_ solutions, the *R*^2^ coefficient spans from 0.9993 to 1.0000, and the AAD of the equilibrium temperatures is from 0.009 to 0.023 K.

As depicted in Figure 2, the measurements obtained for the CO_2_–MeOH–H_2_O system, encompassing methanol concentrations ranging from 0 to 40 mass%, exhibit strong concordance with the data reported by Mohammadi and Richon [59] and Maekawa [39]. However, the equilibrium temperatures of CO_2_ hydrate dissociation at 10 and 20 mass% methanol reported by Robinson and Ng [60,61] are significantly lower (by 1.2–2 K compared with our data and those reported by [39,59]). The observed discrepancies, in conjunction with the pronounced dispersion of equilibrium points on the logarithmic pressure scale, indicate a potential lack of reliability in the experimental data on the equilibrium of CO_2_ hydrate in aqueous methanol solutions by Robinson and Ng [60,61].

Some points measured in [59] at 30 and 40 mass% MeOH are above the vapor–liquid equilibrium line of carbon dioxide. Therefore, they fall within the region of liquid carbon dioxide and describe the three-phase equilibrium L_CO2_–L_w_–H. According to [59], it is evident that the equilibrium line of CO_2_ hydrate for 30 and 40 mass% MeOH in the region above the saturated vapor pressure of CO_2_ has the same slope as at lower pressures (with gaseous carbon dioxide). In the instance of CO_2_ hydrate dissociation into liquid carbon dioxide and water, the volume change ∆*V_dis_* is considerably lower than in the case of decomposition into gaseous carbon dioxide and water. Consequently, the slope of the equilibrium line L_CO2_–L_w_–H, *dP*/*dT* = ∆*H_dis_*/(*T*∆*V_dis_*), is substantially greater than that of V–L_w_–H (see Figure 1 in reference [55] and data from [62,63]). However, the aforementioned authors [59] did not address this issue.

The phase behavior of carbon dioxide hydrate has been previously investigated only at low magnesium chloride concentrations (2–14 mass% MgCl_2_) and temperatures no lower than 267 K, as shown in Figure 3. In contrast, our research collected experimental data on the thermodynamic stability of CO_2_ hydrate at concentrations up to 24.1 mass% MgCl_2_ and temperatures down to 243 K. The results demonstrate that adding MgCl_2_ decreases the thermodynamic activity of water in the solution, thus shifting the equilibrium curves of CO_2_ hydrate to lower temperatures. The equilibrium line for 5.1 mass% MgCl_2_ aligns closely with the data reported by Kang et al. [64] and Zeng et al. [66] for 5 mass% MgCl_2_. Furthermore, the positions of the CO_2_ hydrate equilibrium curves for other magnesium chloride concentrations determined in this study are consistent with the literature data [64,65,66] and complement them.

### 2.2. Comparison of Thermodynamic Inhibition of Carbon Dioxide Hydrate with Methanol and Magnesium Chloride Aqueous Solutions

**Figure 4:**
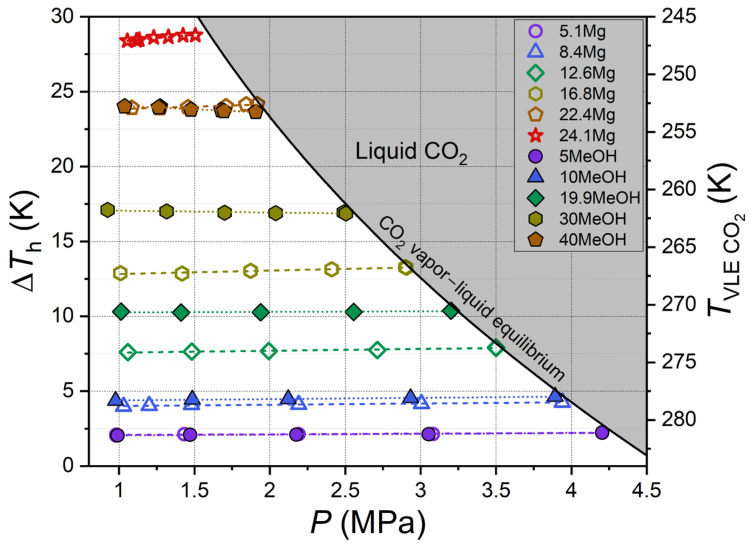
Carbon dioxide hydrate equilibrium temperature suppression ∆*T*_h_ as a function of pressure for aqueous solutions. Color symbols are experimental values, color dashed and dotted lines are linear fits, and black solid line shows vapor–liquid equilibrium for carbon dioxide [56]; legend shows methanol and MgCl_2_ concentration in aqueous solutions in mass%.

To quantitatively compare the thermodynamic inhibitory effect of carbon dioxide hydrate, the equilibrium temperature suppression, herein referred to as Δ*T*_h_, was calculated for each measured equilibrium point. It is defined as the difference between the equilibrium temperatures of CO_2_ hydrate coexisting with pure water (*T*_0_) and a solution of a thermodynamic inhibitor (*T*) at a fixed concentration and pressure. Geometrically, Δ*T*_h_ is the distance between the equilibrium curves in Figure 2 and Figure 3 for gas–water and gas–aqueous inhibitor solution systems at constant pressure. The numerical values of Δ*T*_h_ are tabulated in Tables S1 and S2 for the studied concentrations of MeOH and MgCl_2_. Figure 4 shows how the parameter Δ*T*_h_ depends on pressure for all the samples that were studied.

As shown in Figure 4, hydrate equilibrium temperature suppression is weakly dependent on pressure for aqueous solutions of MeOH and MgCl_2_. For magnesium chloride, the slope *d*Δ*T*_h_/*dP* increases 20.4-fold, rising from 0.044 ± 0.010 K/MPa in the 5.1 Mg sample to 0.897 ± 0.084 K/MPa in the 24.1 Mg sample. For methanol, the slope increases slightly only when transitioning from 5MeOH (0.053 ± 0.011 K/MPa) to 10MeOH (0.084 ± 0.009 K/MPa). Conversely, for more concentrated methanol solutions, the slope decreases and becomes negative for the 30MeOH (−0.122 ± 0.037 K/MPa) and 40MeOH (−0.474 ± 0.058 K/MPa) samples. This variation in behavior is attributed to the differing solubilities of carbon dioxide in aqueous solutions of compounds of varying nature. A case in point is magnesium chloride, which dissociates into ions in an aqueous solution. The solubility of CO_2_ decreases with the increase in salt content due to the salting-out effect [67,68,69]. In contrast, the solubility of CO_2_ in aqueous solutions of nonionic methanol is significantly higher and increases with alcohol concentration [70,71,72]. This can result in a reduction in the MeOH concentration in the solution due to the dissolution of CO_2_. Consequently, there may be a slight decrease in Δ*T*_h_ with an increase in pressure (i.e., an increase in the system’s carbon dioxide content) for the 30MeOH and 40MeOH samples with high alcohol concentrations.

The comparison presented in Figure 4 indicates that low concentrations (5 mass%) of methanol and magnesium chloride exhibit analogous thermodynamic inhibition effects. At elevated concentrations, the anti-hydrate effect of ionic MgCl_2_ surpasses that of nonionic MeOH. Consequently, the concentrations of 40 mass% MeOH and 22.4 mass% MgCl_2_ possess similar values of Δ*T*_h_~24 K. Due to the electrolytic dissociation of MgCl_2_, the total concentration of ions in the solution exceeds the salt concentration. Moreover, salt ions demonstrate a stronger interaction with water dipoles in solution compared with polar methanol molecules. Consequently, given the identical molar and mass contents of MgCl_2_ and MeOH, the thermodynamic activity of water, as well as the ice freezing point, is considerably lower in aqueous solutions of magnesium chloride than in methanol solutions. This assertion is supported by empirical evidence, as demonstrated in both the extant literature [73,74,75,76,77,78,79] and the findings of this study (see Section 2.3).

**Figure 5:**
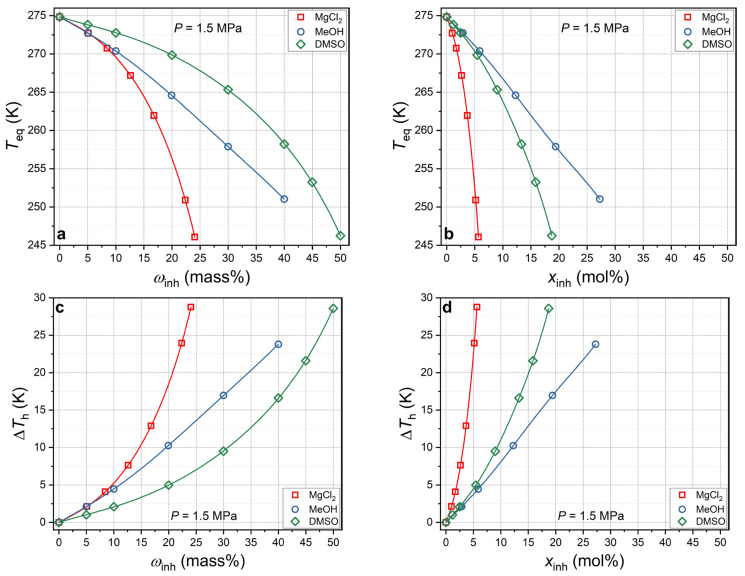
(**a**,**c**) Carbon dioxide hydrate equilibrium temperature *T*_eq_ and suppression of hydrate equilibrium temperature ∆*T*_h_ at a constant pressure of 1.5 MPa as a function of inhibitor concentration in aqueous solution *ω*_inh_ in mass% scale for MeOH and MgCl_2_ (this work) and DMSO [22,48]. Symbols are measured values, and lines are polynomial approximations; (**b**,**d**) the same in mol% scale.

Figure 5 depicts the curves representing the dependence of the equilibrium temperature of CO_2_ hydrate and Δ*T*_h_ at 1.5 MPa on the concentrations of methanol and magnesium chloride, expressed in terms of mass and molar percentages. For the purpose of comparison, curves based on experimental data for dimethyl sulfoxide [22,48] are shown.

The curves for MgCl_2_ and MeOH coincide up to a concentration of ~5.5 mass%. However, at higher mass fractions, magnesium chloride reduces the equilibrium temperature to a greater extent than methanol. Accordingly, at concentrations greater than 5.5 mass%, MgCl_2_ is a stronger THI than MeOH. On a mass percentage scale, dimethyl sulfoxide (DMSO) is a weaker THI than methanol (MeOH) at concentrations up to 40 mass%. This outcome is attributable to a more pronounced decline in the thermodynamic activity of water (ice freezing point) at a constant mass fraction of methanol in an aqueous solution, compared with DMSO within the specified concentration range. This assertion is corroborated by experimental data [76,77,80,81,82]. The lower molar mass of methanol (32.04 g/mol) compared with DMSO (78.13 g/mol) is a contributing factor in this regard. If the mass fraction remains constant in the aqueous solution, the molar fraction of methanol will exceed that of DMSO. However, the inhibitory effect of DMSO with regard to CO_2_ hydrate increases more rapidly than that of MeOH, particularly at elevated concentrations (>30 mass%). A similar phenomenon occurs in the inhibition of methane hydrate [82,83] and is associated with greater deviation from the ideal behavior of DMSO aqueous solutions than of MeOH aqueous solutions.

Transitioning from the mass% scale to the mol% scale (see Figure 5b,d) results in an increase in the difference in inhibitory properties between MeOH and MgCl_2_. This increase is attributable to the electrolytic dissociation of MgCl_2_ in an aqueous solution, which results in a higher total molar fraction of particles (ions) present in the system. The higher non-ideality of DMSO aqueous solutions compared with MeOH explains the more pronounced inhibitory effect of dimethyl sulfoxide compared with MeOH on a mol% scale.

**Figure 6:**
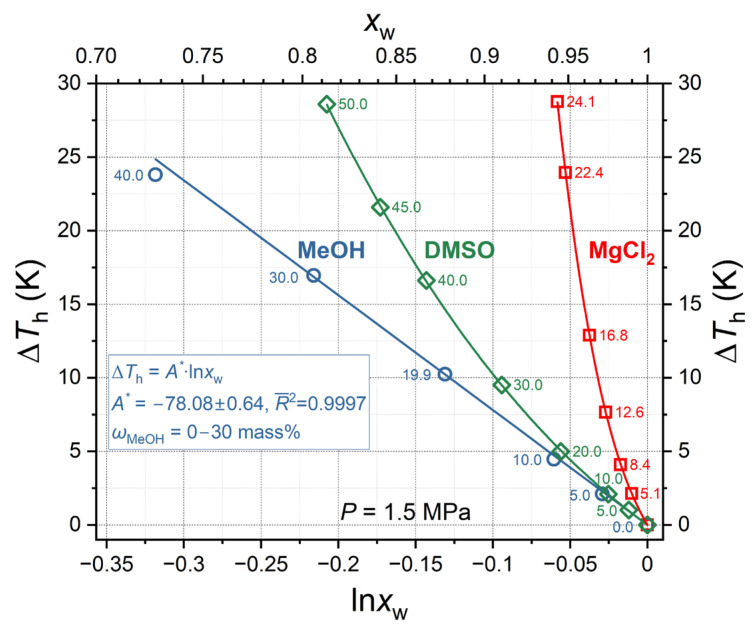
Carbon dioxide hydrate equilibrium temperature suppression ∆*T*_h_ as a function of water mole fraction *x*_w_ in aqueous solution and the same value in logarithm scale for MeOH and MgCl_2_ (this work) and DMSO [22,48]. Symbols are measured values, straight line for MeOH is a linear fit, curves for DMSO and MgCl_2_ represent cubic fits, and the numerical values show the mass percentage of solutes.

Equation (2) relates the value of Δ*T*_h_ to the thermodynamic activity of water *a*_w_ in the solution:

(2) $$\Delta T_{h}=A\cdot \ln a_{w},$$

where *A* is the proportionality coefficient. Equation (2) can be written in an approximate form by replacing the value *a*_w_ with the molar fraction of water in solution *x*_w_:

(3) $$\Delta T_{h}=A^{*}\cdot \ln x_{w},$$

where A* is the proportionality coefficient. Figure 6 displays the correlation between Δ*T*_h_ and ln *x*_w_ for MeOH and MgCl_2_ (this work) and DMSO (data from [22,48]) at a fixed pressure of 1.5 MPa.

As illustrated in Figure 6, the linear correlation in equation 3 is only well observed for aqueous MeOH solutions (*R*^2^ = 0.9997) in the range of 0–30 mass%, with a slope parameter A* = −78.08 ± 0.64 K. The dependence of Δ*T*_h_ on ln *x*_w_ for DMSO up to 10 mass% coincides with the straight line for methanol. A rapid nonlinear increase in Δ*T*_h_ is noticeable when DMSO contents exceed 10 mass%. This increase can be accurately described by a cubic polynomial, with a correlation coefficient *R*^2^ = 0.99996. In the case of magnesium chloride, a decrease in the molar fraction of water in the solution is associated with the most pronounced increase in Δ*T*_h_. This increase can be described by a nonlinear model within the studied concentration range, such as a third-degree polynomial (*R*^2^ = 0.99999).

**Figure 7:**
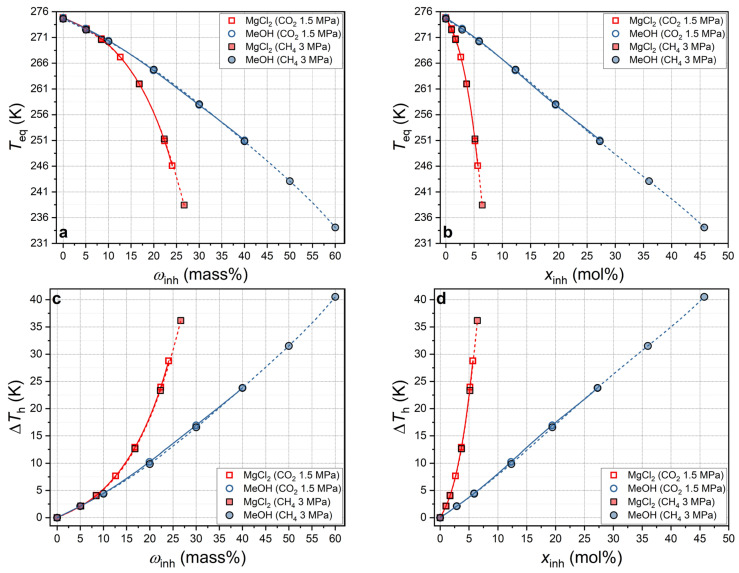
(**a**,**c**) Carbon dioxide and methane hydrate equilibrium temperature and suppression of hydrate equilibrium temperature as a function of MeOH and MgCl_2_ concentration in aqueous solution in mass% scale (CO_2_ at 1.5 MPa—this work; CH_4_ at 3 MPa—data from [78,79]). Symbols are measured values, and lines are polynomial approximations; (**b**,**d**) the same in mol% scale.

The inhibitory capacity of methanol and magnesium chloride with respect to carbon dioxide was compared with that of methane [78,79]. Methane and carbon dioxide are chemically distinct gases that form thermodynamically stable sI hydrates. Figure 7 provides a visual representation of the relationship between the equilibrium temperatures of CH_4_ hydrate (V–L_w_–H at 3 MPa) and CO_2_ hydrate (V–L_w_–H at 1.5 MPa) and the value of Δ*T*_h_ as a function of the concentration of thermodynamic inhibitors in solution, expressed in terms of mass% (panels a and c) and mol% (panels b and d).

**Figure 10:**
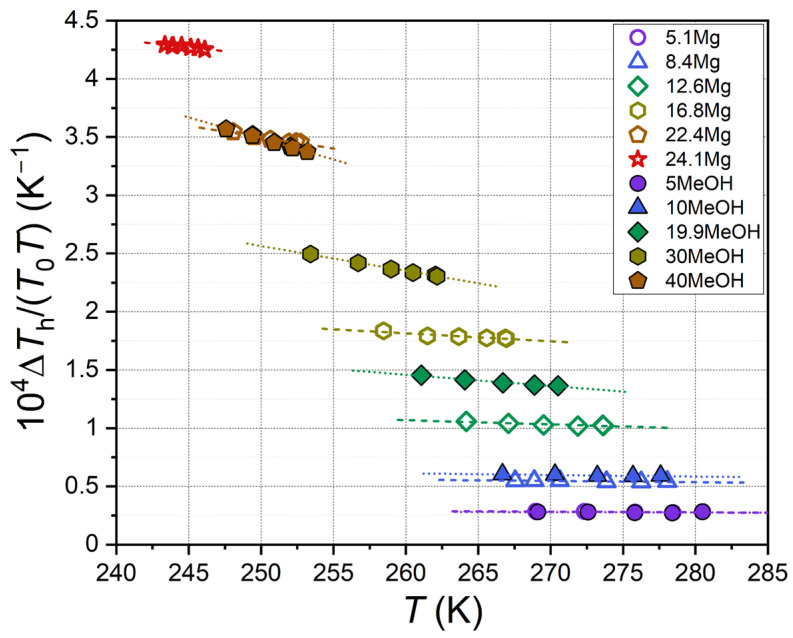
Parameter Δ*T*_h_/(*T*_0_*T*) for studied samples of MeOH and MgCl_2_ aqueous solutions vs. carbon dioxide hydrate equilibrium temperature. Symbols are equilibrium points, and dashed and dotted lines are linear fits; legend shows MeOH and MgCl_2_ concentration in aqueous solutions in mass%.

Figure 7 indicates that the curves for CO_2_ and CH_4_ are closely aligned, thereby demonstrating a high degree of agreement. A notable observation is the slightly more pronounced decrease in equilibrium temperature for CO_2_ hydrate compared with CH_4_, at a constant concentration of MeOH or MgCl_2_. These findings are consistent with a similar comparison reported for DMSO (see Figure 10 of reference [22]). For instance, for a sample containing 22.4 mass% MgCl_2_, Δ*T*_h_ = 23.95 K (1.5 MPa) for CO_2_ and Δ*T*_h_ = 23.54 K (3 MPa) for CH_4_. Analogously, for a sample containing 30 mass% MeOH, Δ*T*_h_ = 16.95 K (1.5 MPa) for CO_2_ and Δ*T*_h_ = 16.57 K (3 MPa) for CH_4_.

### 2.3. Ice Freezing Temperatures for Methanol and Magnesium Chloride Aqueous Solutions

When a thermodynamic inhibitor is introduced into a gas–water system, the temperature of the three-phase equilibrium V–L_w_–H decreases at constant pressure. This phenomenon can be attributed to a decrease in the thermodynamic activity of H_2_O in the solution relative to the pure solvent. A similar phenomenon occurs in the case of the two-phase equilibrium ice–aqueous solution. The melting point of ice *T*_ice_ decreases in an antifreeze solution compared with pure solvent. To analyze the proportionality of Δ*T*_h_ and Δ*T*_ice_, the ice freezing temperatures were measured at an atmospheric pressure of 0.1 MPa for aqueous solutions of MeOH and MgCl_2_. To ascertain the ice onset and peak ice crystallization temperatures (*T*_ice onset_ and *T*_ice peak_, respectively), three repetitions were conducted at a linear cooling rate of 10 K/h, under an intensive stirring of the aqueous solutions. The melting temperature of the last ice crystals, *T*_ice melt_, was also measured using a 5 K/h heating ramp under intensive stirring. The numerical values of these quantities are presented in Tables S5 and S6 for aqueous solutions of methanol and magnesium chloride, respectively. Figures S3–S5 present examples of the thermal curves employed to ascertain the crystallization and melting temperatures of ice. It is well established that the process of crystallization of the solid phase from a supersaturated solution requires a supercooling relative to the equilibrium temperature. Consequently, for the purposes of analysis and correlation, we used *T*_ice melt_ (hereafter *T*_ice_) values more closely aligned with the equilibrium freezing temperature of ice in aqueous solutions.

**Figure 8:**
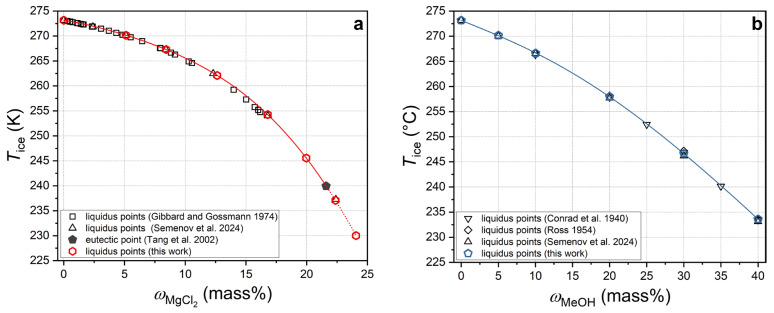
(**a**) Liquidus for MgCl_2_–H_2_O system at 0.1 MPa, where red hexagons show measured ice melting temperatures of this work, black pentagon represents eutectic point from [85], black triangles show ice peak crystallization temperatures from [78,79], black squares show literature data from [75], red solid curve shows liquidus line approximated with polynomial from data of this work (stable ice–aqueous solution equilibrium), and red dotted curve shows metastable continuation (metastable ice–supercooled aqueous solution equilibrium) of liquidus line approximated with polynomial from data of this work. (**b**) Liquidus MeOH–H_2_O system at 0.1 MPa, where blue pentagons show measured ice melting temperatures of this work, black triangles show ice peak crystallization temperatures from [78,79], black diamonds and down triangles show literature data from [77,84], and blue solid curve shows liquidus line approximated with polynomial from data of this work.

Figure 8 depicts the measured ice freezing temperatures for aqueous solutions as a function of the MeOH and MgCl_2_ concentrations, in comparison with literature data for methanol [77,78,79,84] and magnesium chloride [75,78,79,85].

The findings of this study concur with the extant data in the literature. In previous works [78,79], the freezing point of ice was measured with the same equipment but a different method: the temperature was decreased in small steps (0.5–1 K). The freezing point of the solution in [78,79] was taken as the *T*_ice peak_, which may be underestimated relative to the equilibrium, especially in concentrated solutions, which are more prone to supercooling. The difference between the *T*_ice peak_ values measured in [78,79] and the *T*_ice melt_ values in this study increases as the antifreeze component content in the solution increases. Nevertheless, the observed difference was found to be relatively negligible (0.05–0.5 K) within the concentration range examined.

The crystallization and melting temperatures of the metastable ice phase were measured for aqueous solutions of 22.37 and 24.06 mass% MgCl_2_. For the MgCl_2_–H_2_O system at 0.1 MPa, the eutectic composition contains 21.6 mass% MgCl_2_ and melts at 239.95 K (non-variant equilibrium of an aqueous salt solution, ice, and MgCl_2_·12H_2_O crystalline hydrate solid) [85]. Therefore, the thermodynamically stable solid phase for 22.37 and 24.06 mass% aqueous MgCl_2_ solutions is MgCl_2_·12H_2_O, which crystallizes at higher supercooling and melts at higher temperatures than metastable ice. Figures S6 and S7 offer a visual representation of the thermal curves associated with the processes of crystallization and melting of metastable ice, as well as thermodynamically stable crystalline hydrate MgCl_2_·12H_2_O.

## 3. Discussion

### 3.1. Thermodynamic Consistency of Hydrate Equilibrium Data

**Figure 9:**
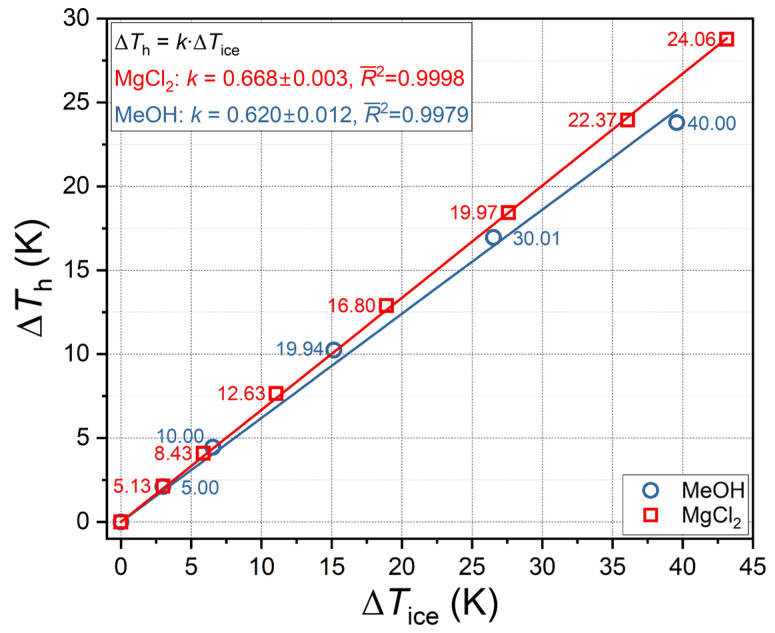
Linear correlation between suppression of carbon dioxide hydrate equilibrium temperature (at 1.5 MPa) and ice melting temperature (at 0.1 MPa) for aqueous MeOH and MgCl_2_ solutions. Symbols are experimental points; solid lines are linear fits; the numerical values show the mass percentage of solutes.

The thermodynamic consistency of the experimental data was evaluated based on the results obtained from analyzing the proportionality between the values of Δ*T*_h_ and Δ*T*_ice_ for the aqueous solutions studied. For aqueous solutions of compounds exhibiting thermodynamic inhibitor properties, a direct proportionality between the values of Δ*T*_h_ and Δ*T*_ice_ is to be expected. This relationship can be attributed to the fact that the suppression in the equilibrium ice melting temperature and gas hydrate dissociation temperature are consequences of a single physicochemical phenomenon, namely, the decrease in the thermodynamic activity of water when an inhibitor (antifreeze component) is added to it. The results of the analysis are displayed in Figure 9.

As can be seen, these values are linearly related (*R*^2^ = 0.9998 for MgCl_2_; *R*^2^ = 0.9979 for MeOH) and described by equation 4 with a single coefficient, *k*:

(4) $$\Delta T_{h}=k\cdot \Delta T_{ice}$$

Intriguingly, over a broad range of concentrations, the slope coefficient for MgCl_2_ is slightly greater than that for MeOH: 0.668 ± 0.003 and 0.620 ± 0.012, respectively. Concurrently, within the Δ*T*_h_ range of 10 K, all data points align on a single linear trajectory for MgCl_2_. Conversely, at elevated MeOH concentrations, with the associated increase in the Δ*T*_h_ values, the anti-hydrate effect of methanol increases more gradually. This is presumably due to the dissolution of carbon dioxide. As previously discussed in Section 2.2, the solubility of carbon dioxide in aqueous solutions of methanol [70,71,72] and magnesium chloride [67,68,69] varies significantly. In the instance of methanol, the solubility of CO_2_ increases concomitantly with the increase in alcohol concentration. In the case of magnesium chloride, the salting-out effect leads to a decrease in the solubility of CO_2_ as the salt content rises. Accordingly, in the event of high feed methanol concentrations (30 and 40 mass%), the intensive dissolution of carbon dioxide will result in a decrease in the equilibrium concentration of methanol compared with the initial concentration. This will result in a slower increase in Δ*T*_h_ for high MeOH concentrations, as observed in Figure 9.

To evaluate thermodynamic consistency, the approach described in [86,87,88,89,90] was also tested. The authors of these works proposed analyzing the change in the parameter ∆*T*_h_/(*T*_0_*T*) from the hydrate equilibrium temperature in a solution containing a thermodynamic inhibitor. Their assertion is that this parameter is independent of *T* and thus can be adequately described by a linear equation with a zero slope for each concentration. Figure 10 presents the outcomes resulting from the implementation of the aforementioned approach.

The linear approximations and analysis of variance (ANOVA) indicate that the slope parameter is statistically insignificant from 0 only for diluted solutions (5.1Mg, 8.4Mg, 5MeOH, and 10MeOH). However, for more concentrated aqueous solutions, the slope parameter of the approximating linear regression model is statistically significant and increases in magnitude (modulo) with the increase in inhibitor concentration. The findings presented in this study, derived from the approach outlined in [86,87,88,89,90], demonstrate a strong congruence with our prior observations concerning other hydrate-forming gases and thermodynamic inhibitors [22,58,78,79,83,91,92,93]. These findings imply that the application of the parameter ∆*T*_h_/(*T*_0_*T*) for the evaluation of the thermodynamic consistency of gas hydrate equilibrium data is inadequate, particularly at elevated inhibitor concentrations, when inhibitors crystallize from solution (e.g., urea [93]), or when an aqueous inhibitor solution undergoes phase separation into two liquid phases (e.g., 2-butoxyethanol [58]). The underlying rationales for these findings are discussed in more detail in the referenced literature [78,91].

### 3.2. Results of PXRD Analysis

**Figure 11:**
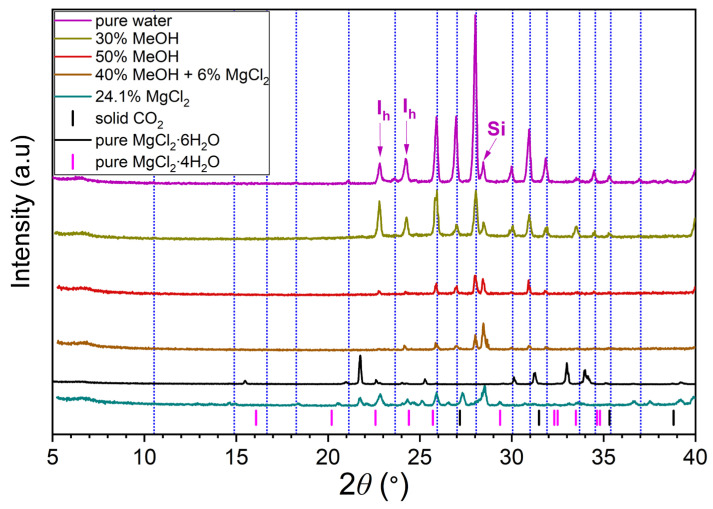
PXRD patterns of hydrate samples obtained in the CO_2_–MeOH–H_2_O, CO_2_–MgCl_2_–H_2_O, and CO_2_–MeOH–MgCl_2_–H_2_O systems. The vertical blue dotted lines indicate the positions of the CO_2_ hydrate diffraction peaks; the PXRD pattern of pure magnesium chloride hexahydrate and the peak positions of magnesium chloride tetrahydrate are shown for comparison. Silicon (Si) was used as an internal standard. The legend shows MeOH and MgCl_2_ concentration in aqueous solutions in mass%.

Figure 11 shows the resulting PXRD patterns.

The analysis results indicate that the incorporation of methanol into the large cavities of the sI hydrate framework is unlikely. The unit cell parameter of carbon dioxide hydrate was determined to be 11.86 ± 0.04 Å in all samples at 133 K, which is consistent with literature data for sI hydrate [94]. Additionally, the 24.1 mass% MgCl_2_ sample was found to contain solid CO_2_ and a mixture of magnesium chloride crystalline hydrates, most likely MgCl_2_·4H_2_O and MgCl_2_·6H_2_O. The absence of solid CO_2_ in the 30 mass% and 50 mass% methanol samples is attributed to the high solubility of CO_2_ in cold water–methanol solutions.

## 4. Materials and Methods

This study used the following materials: high-purity carbon dioxide (CO_2_; 99.99 vol%; NIIKM, Moscow, Russia), chemically pure methanol (MeOH; 99.9 mass%; Vekton, Saint Petersburg, Russia), anhydrous magnesium chloride (MgCl_2_; 99.21 mass%; Alfa Aesar, Landsmeer, The Netherlands), and distilled water obtained in the laboratory. A series of aqueous solutions of methanol and magnesium chloride at a given concentration were prepared gravimetrically in amounts of at least 350 g using PA413C scales (Ohaus, Parsippany, NY, USA) with a resolution of 0.001 g and a maximum error of ±0.01 g. The uncertainty in the concentrations of methanol and magnesium chloride in the resulting solutions did not exceed 0.02 mass%.

**Figure 12:**
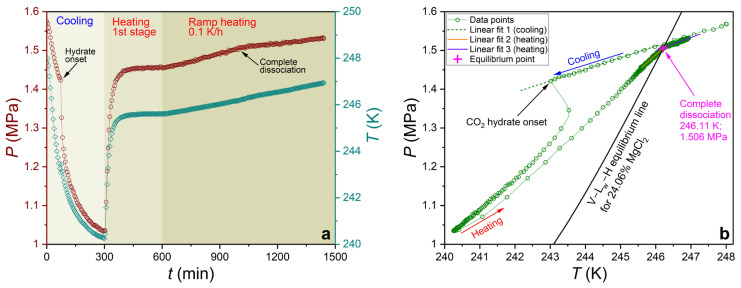
(**a**) Experimental pressure and temperature curves versus time for a 24.06 mass% aqueous MgCl_2_ solution, obtained using the 0.1 K/h ramp heating technique; (**b**) pressure–temperature trace and the result of determining the equilibrium point for this experiment.

**Figure 13:**
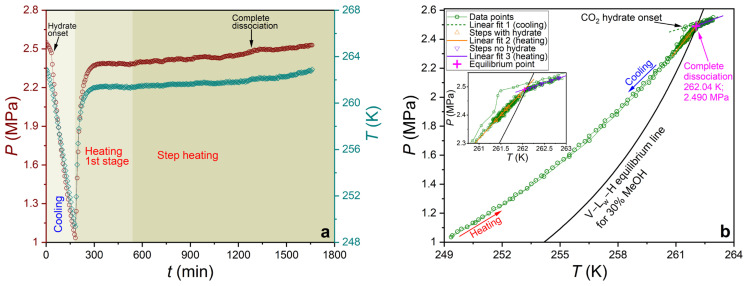
(**a**) Experimental pressure and temperature curves versus time for a 30 mass% aqueous MeOH solution, obtained using the step heating technique; (**b**) pressure–temperature trace and the result of determining the equilibrium point for this experiment.

The phase equilibrium of carbon dioxide hydrate was measured using a GHA350 apparatus (PSL Systemtechnik, Osterode am Harz, Germany). A thorough description of the apparatus and the procedure for calibrating pressure/temperature sensors can be found in previous works [83,95]. For the present experiment, 300 mL of the prepared aqueous solution was placed in an autoclave. The equilibrium conditions of the CO_2_ hydrate were measured following the 0.1 K/h ramp heating and step heating techniques previously analyzed [96]. For the systems examined in this study, both methods yielded equilibrium temperature and pressure values that did not differ from each other by more than the maximum measurement error (±0.1 K and ±0.017 MPa, respectively). Figure 12 and Figure 13 illustrate the experimental curves obtained and the results of determining the coordinates of the equilibrium point (complete dissociation of CO_2_ hydrate) for the 0.1 K/h ramp heating technique and the step heating technique, respectively.

The ice crystallization and melting temperatures of methanol and magnesium chloride aqueous solutions were measured using a calibrated PRT 5622–10-P temperature sensor (Fluke, Everett, WA, USA) in tandem with a 1524 reference thermometer (Fluke, Everett, WA, USA), which has a maximum error of ±0.04 K. Further information regarding the equipment and measurement methods can be found elsewhere [58].

The phase composition of CO_2_ hydrate samples obtained from aqueous solutions of methanol and magnesium chloride was studied using powder X-ray diffraction. The measurements were conducted using a D8 Advance diffractometer (Bruker, Ettlingen, Germany) in 2θ scanning mode within the angular range of 5° to 42° at 133 K (Cu Ka X-ray source with a wavelength of 1.5418 Å). To obtain more accurate data, silicon (Si), whose reflex position is known with high precision, was added to each sample as an internal standard.

A series of CO_2_ hydrate samples was obtained from different solutions to identify the hydrate type. Pure water was selected as the reference sample. The phase state of CO_2_ hydrate obtained from four distinct aqueous solutions was studied: 30 mass% MeOH, 50 mass% MeOH, 24.1 mass% MgCl_2_, and a mixed solution of 40 mass% MeOH and 6 mass% MgCl_2_. According to phase equilibrium data [78,79], the latter three solutions have been shown to possess the same anti-hydrate properties. Carbon dioxide hydrate was obtained from frozen, ground aqueous solutions. For pure water, hydrate synthesis was performed at 274.15 K and 3.5 MPa. For the 30 mass% MeOH sample, the synthesis occurred at 248.15 K and 1.5 MPa. For 50 mass% MeOH, 24.1 mass% MgCl_2_, and 40 mass% MeOH + 6 mass% MgCl_2_, the synthesis occurred at 233.15 K and 1 MPa. The pressure was selected in proximity to the CO_2_ condensation line within the gas phase region. The frozen solutions were thoroughly ground and subsequently placed in a precooled autoclave (at 274.15, 248.15, or 233.15 K, depending on the system). Carbon dioxide was then introduced into the cell to displace the air. The cell was pressurized with CO_2_ to the specified pressure, and it was maintained for 24 h under isothermal–isochoric conditions. Prior to the removal of the resulting samples, the autoclave was cooled to the boiling point of liquid nitrogen. The resulting samples were meticulously ground in an aluminum mortar under liquid nitrogen, mixed with silicon powder, and transferred with a spatula to the diffractometer sample holder, which had been cooled to 133 K.

## 5. Conclusions

This paper explores the thermodynamic stability of carbon dioxide hydrate in aqueous solutions of magnesium chloride and methanol across a wide concentration range. New experimental data on the three-phase equilibrium (V–L_w_–H) were obtained at salt concentrations up to 24.1 mass% MgCl_2_ and temperatures down to 243 K. Methanol and magnesium chloride are thermodynamic inhibitors of CO_2_ hydrate formation because an increase in their concentration in a solution lowers the equilibrium temperature. A detailed analysis of the anti-hydrate effect of MeOH and MgCl_2_ was performed. It was demonstrated that on a mass percentage scale, the anti-hydrate effect of MeOH and MgCl_2_ does not differ by up to 5 mass%. However, at higher concentrations, ionic magnesium chloride is a stronger THI than nonionic methanol. The anti-hydrate effect of methanol Δ*T*_h_ was found to be linearly related to the natural logarithm of the molar fraction of water in solution over a sufficiently wide range. For magnesium chloride, however, a similar dependence is nonlinear and can be well described by a cubic polynomial. The inhibitory effects of methanol and magnesium chloride on carbon dioxide and methane hydrates were compared. It has been established that the inhibitory effects of MeOH and MgCl_2_ are similar for hydrate-forming gases of different chemical nature. In order to verify the thermodynamic consistency of the data, the ice freezing temperatures of the aqueous solutions were measured at 0.1 MPa. Linear correlations between Δ*T*_h_ and Δ*T*_ice_ were obtained for MeOH and MgCl_2_, thereby confirming thermodynamic consistency. The phase composition of CO_2_ hydrate samples was investigated using PXRD at 133 K. These samples were synthesized from aqueous solutions of methanol and magnesium chloride. The formation of sI hydrate with a unit cell parameter of 11.86 ± 0.04 Å was found in all cases, which is consistent with literature data for pure carbon dioxide hydrate.

The findings of this study suggest the potential of magnesium chloride as a thermodynamic inhibitor of gas hydrates, exhibiting a more pronounced inhibitory effect compared with methanol (industrial THI). Magnesium chloride possesses several noteworthy advantages, including non-volatility, non-flammability, and environmental safety. Additionally, it is readily available due to its prevalence in nature as the mineral bischofite.

## Data Availability

The original contributions presented in this study are included in the article and Supplementary Materials. Further inquiries can be directed to the corresponding authors.

## Supplementary Materials

The following supporting information can be downloaded at: https://www.mdpi.com/article/10.3390/ijms27041792/s1.
