# Supplementary material for: Thermodynamic Inhibition of Carbon Dioxide Hydrate with Magnesium Chloride and Methanol: Comparative Phase Equilibrium and PXRD Study
Source: Int J Mol Sci. 2026 Feb 13;27(4):1792. doi: 10.3390/ijms27041792 (PMC12941068; doi:10.3390/ijms27041792)
Supplement: Supplementary file 1 [file ijms-27-01792-s001.zip › ijms-4102362-supplementary.pdf]

Thermodynamic Inhibition of Carbon Dioxide Hydrate with Magnesium Chloride and Methanol: Comparative Phase Equilibrium and PXRD Study

Anton Semenov [1,*], Rais Mendgaziev [1], Andrey Stoporev [1,2], Timur Tulegenov [1], Daniil Lednev [1], Murtazali Yarakhmedov [1], Vladimir Istomin [1], Daria Sergeeva [1,3] and Rawil Fakhrullin [4,*]

[1] *Department of Physical and Colloid Chemistry, Gubkin University, 65, Leninsky Prospekt, Building 1, 119991 Moscow, Russia*

[2] *Moscow Center for Advanced Studies, Kulakova Str. 20, 123592 Moscow, Russia*

[3] *Center for Petroleum Science and Engineering, Skolkovo Institute of Science and Technology (Skoltech), Bolshoy Boulevard, 30, Building 1, 121205 Moscow, Russia*

[4] *Institute of Fundamental Medicine and Biology, Kazan Federal University, 420008 Kazan, Russia*

* *Correspondence: semenov.a@gubkin.ru (A.S.); kazanbio@gmail.com (R.F.)*

Table S1. Carbon dioxide hydrate dissociation data for CO_2–MeOH–H_2O system (equilibrium temperature T, pressure P, and hydrate equilibrium temperature suppression ΔT_h at P value relative to the system with pure H_2O (sample #1)).

Sample #	MeOH feed concentration in aqueous solution, mass% [a] (mol%)	Point #	T_{eq}, K [b]	P_{eq}, MPa [c]	ΔT_h, K
1	0	1	271.60 [d]	1.044 [d]	0
		2	272.37	1.124	0
		3	272.35	1.123	0
		4	273.74	1.325	0
		5	275.08	1.545	0
		6	276.58	1.849	0
		7	278.17	2.247	0
		8	279.70	2.731	0
		9	280.79	3.165	0
		10	281.99	3.755	0
		11	283.16	4.523	0
2	5.00 (2.87)	12	269.12	0.990	2.05
		13	272.58	1.471	2.09
		14	275.81	2.176	2.09
		15	278.41	3.053	2.12
		16	280.49	4.204	2.24
3	10.00 (5.88)	17	266.68	0.977	4.36
		18	270.30	1.485	4.45
		19	273.23	2.122	4.47
		20	275.69	2.934	4.54
		21	277.60	3.891	4.64
4	19.94 (12.28)	22	261.07	1.013	10.31
		23	264.07	1.411	10.25
		24	266.71	1.940	10.27
		25	268.89	2.556	10.29
		26	270.51	3.202	10.37
5	30.01 (19.42)	27	253.42	0.924	17.10
		28	256.71	1.315	17.00
		29	258.98	1.700	16.91
		30	260.50	2.039	16.88
		31	262.04	2.490	16.93
		32	262.16	2.505	16.86
6	40.00 (27.26)	33	247.59	1.037	24.00
		34	249.42	1.272	24.00
		35	249.40	1.262	23.95
		36	250.91	1.476	23.79
		37	252.03	1.678	23.75
		38	252.17	1.695	23.70
		39	253.20	1.907	23.64

[a] Expanded uncertainty in the concentrations of MeOH does not exceed 0.02 mass%.
[b] Expanded uncertainty is 0.1 K ($k = 2$)
[c] Expanded uncertainty is 0.02 MPa ($k = 2$)
[d] Lower quadruple point Q_1 (four-phase V–L_w–I–H equilibrium) for CO_2–H_2O system measured in [1]

Table S2. Carbon dioxide hydrate dissociation data for CO_2–$MgCl_2$–H_2O system (equilibrium temperature *T*, pressure *P*, and hydrate equilibrium temperature suppression ΔT_h at *P* value relative to the system with pure H_2O (sample #1)).

Sample #	$MgCl_2$ feed concentration in aqueous solution, mass% [a] (mol%)	Point #	T_{eq}, K [b]	P_{eq}, MPa [c]	ΔT_h, K
1	0	1	271.60 [d]	1.044 [d]	0
		2	272.37	1.124	0
		3	272.35	1.123	0
		4	273.74	1.325	0
		5	275.08	1.545	0
		6	276.58	1.849	0
		7	278.17	2.247	0
		8	279.70	2.731	0
		9	280.79	3.165	0
		10	281.99	3.755	0
		11	283.16	4.523	0
2	5.13 (1.01)	12	269.05	0.982	2.04
		13	272.33	1.433	2.12
		14	275.83	2.191	2.13
		15	278.46	3.081	2.14
		16	280.50	4.205	2.23
3	8.43 (1.71)	17	267.55	1.030	3.98
		18	268.87	1.200	4.03
		19	270.63	1.480	4.09
		20	273.85	2.191	4.11
		21	276.25	3.005	4.16
		22	278.06	3.939	4.26
4	12.63 (2.66)	23	264.18	1.059	7.60
		24	267.09	1.482	7.65
		25	269.52	1.994	7.68
		26	271.89	2.713	7.75
		27	273.61	3.500	7.91
		28	273.64	3.501	7.88
5	16.80 (3.68)	29	258.47	1.009	12.87
		30	261.50	1.418	12.86
		31	263.66	1.872	13.03
		32	265.58	2.410	13.14
		33	266.87	2.900	13.27
		34	266.93	2.905	13.23
6	22.37 (5.17)	35	248.08	1.084	23.91
		36	249.52	1.271	23.89
		37	250.64	1.457	23.95
		38	251.92	1.709	24.01
		39	252.42	1.843	24.14
		40	252.72	1.917	24.16
7	24.06 (5.66)	41	243.36	1.055	28.39
		42	243.84	1.116	28.42
		43	243.93	1.133	28.46
		44	244.51	1.229	28.61
		45	245.14	1.327	28.65
		46	245.65	1.425	28.75
		47	246.11	1.506	28.76

[a] Expanded uncertainty in the concentrations of $MgCl_2$ does not exceed 0.02 mass%.
[b] Expanded uncertainty is 0.1 K ($k = 2$)
[c] Expanded uncertainty is 0.02 MPa ($k = 2$)
[d] Lower quadruple point Q_1 (four-phase V–L_w–I–H equilibrium) for CO_2–H_2O system measured in [1]

Table S3. The coefficients of the equation 1 describing the variation in the equilibrium temperature T (K) of CO_2 hydrate as a function of pressure P for 0–40 mass% (0–27.26 mol%) aqueous methanol (MeOH) solutions.

MeOH feed concentration, mass% (mol%)	a_1	a_2	a_3	b_1	Adj. R^2	Average absolute deviation of eq. T, K
0	0.17424	289.40066	289.27018	1.13358	0.99991	0.023
5.00 (2.87)	0.01755	288.11873	301.65373	1.19081	0.99994	0.013
10.00 (5.88)	0.19369	284.27329	263.91731	1.05478	0.99999	0.004
19.94 (12.28)	0.61601	275.16500	159.15466	0.66672	0.99993	0.012
30.01 (19.42)	0.47358	269.66989	172.53197	0.74166	0.99988	0.013
40.00 (27.26)	-28.89937	427.87925	3037.08696	12.89603	0.99968	0.016

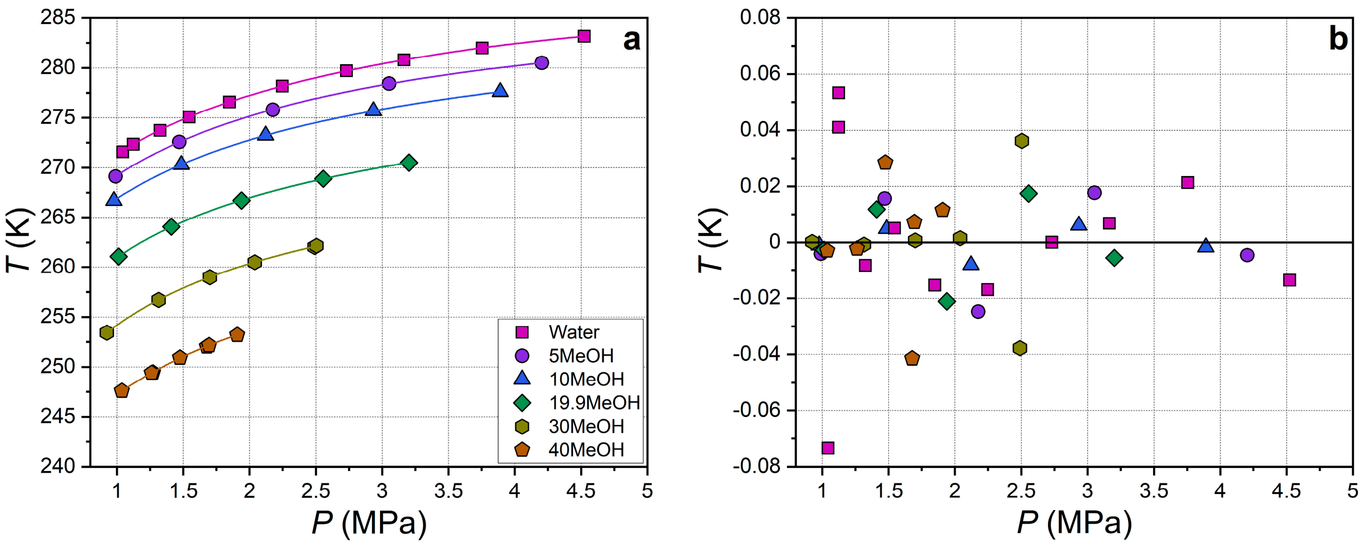

Figure S1. **(a)** Measured equilibrium temperatures and pressures of CO_2 hydrate for aqueous MeOH solutions with different concentrations (symbols), with lines showing the results of approximating experimental points (equation 1 and Table S3); **(b)** difference between the experimental and approximate values of the equilibrium temperature as a function of pressure.

Table S4. The coefficients of the equation 1 describing the variation in the equilibrium temperature T (K) of CO_2 hydrate as a function of pressure P for 0–24.06 mass% (0–5.66 mol%) aqueous $MgCl_2$ solutions.

$MgCl_2$ feed concentration, mass% (mol%)	a_1	a_2	a_3	b_1	Adj. R^2	Average absolute deviation of eq. T, K
0	0.17424	289.40066	289.27018	1.13358	0.99991	0.023
5.13 (1.01)	0.09042	287.64376	291.99436	1.15365	0.99993	0.022
8.43 (1.71)	0.04433	286.04635	313.19619	1.24191	0.99998	0.009
12.63 (2.66)	0.08450	281.50605	266.25260	1.07764	0.99997	0.011
16.80 (3.68)	1.23263	267.55207	46.21192	0.21909	0.99989	0.018
22.37 (5.17)	-0.65892	264.42018	148.35850	0.66629	0.99978	0.014
24.06 (5.66)	5.15640	234.17862	-201.65894	-0.84487	0.99925	0.015

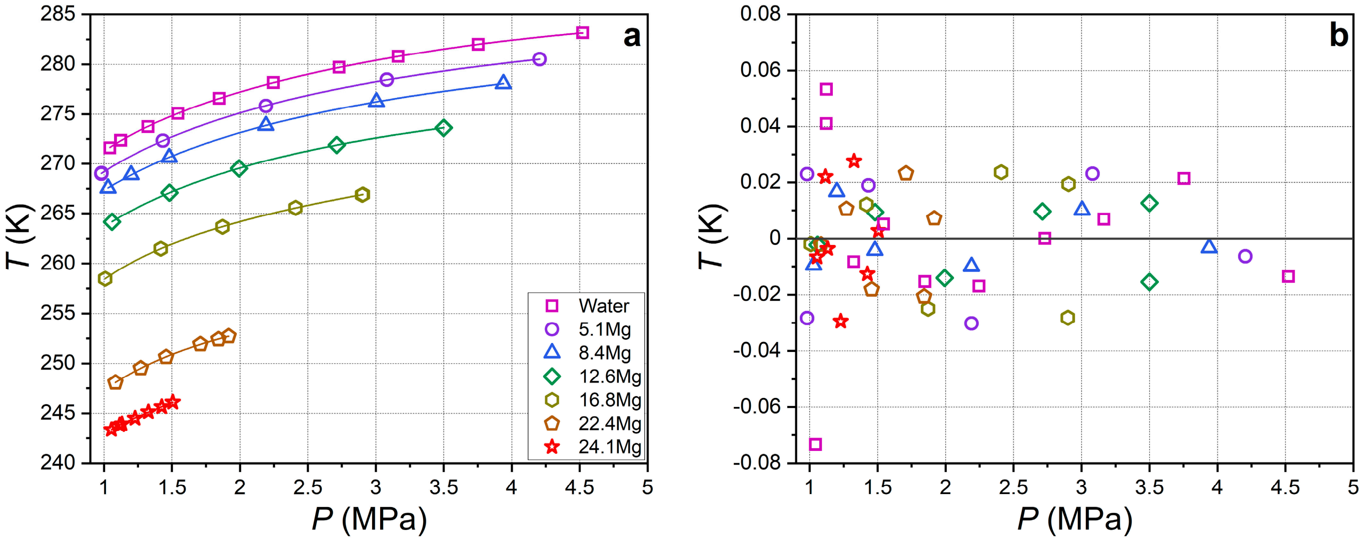

Figure S2. **(a)** Measured equilibrium temperatures and pressures of CO_2 hydrate for aqueous $MgCl_2$ solutions with different concentrations (symbols), with lines showing the results of approximating experimental points (equation 1 and Table S4); **(b)** difference between the experimental and approximate values of the equilibrium temperature as a function of pressure.

Table S5. Experimental data on ice freezing and melting temperatures for aqueous MeOH solutions; mean ± standard deviation values for three replicates are shown in bold.

MeOH concentration, mass% (mol%)	$T_{\text{ice onset}}$, K	$T_{\text{ice peak}}$, K	$T_{\text{ice melt}}$, K
0 (0)	271.01	273.16	273.16
	272.38	273.16	273.16
	272.36	273.16	273.15
	**271.92±0.79**	**273.16±0.00**	**273.16±0.01**
5.00 (2.87)	268.98	270.04	270.17
	268.94	270.03	270.17
	268.42	270.01	270.17
	**268.78±0.32**	**270.03±0.02**	**270.17±0.00**
10.03 (5.90)	265.32	266.39	266.61
	264.66	266.32	266.61
	264.00	266.25	266.60
	**264.66±0.66**	**266.32±0.07**	**266.60±0.00**
20.00 (12.32)	254.59	257.04	257.97
	255.63	257.30	257.97
	254.33	256.97	257.98
	**254.85±0.69**	**257.10±0.17**	**257.97±0.01**
30.00 (19.41)	243.14	245.12	246.66
	241.80	244.62	246.61
	243.49	245.24	246.63
	**242.81±0.89**	**244.99±0.33**	**246.63±0.03**
40.00 (27.27)	230.01	231.54	233.63
	229.51	231.29	233.60
	228.53	230.86	233.52
	**229.35±0.75**	**231.23±0.34**	**233.58±0.06**

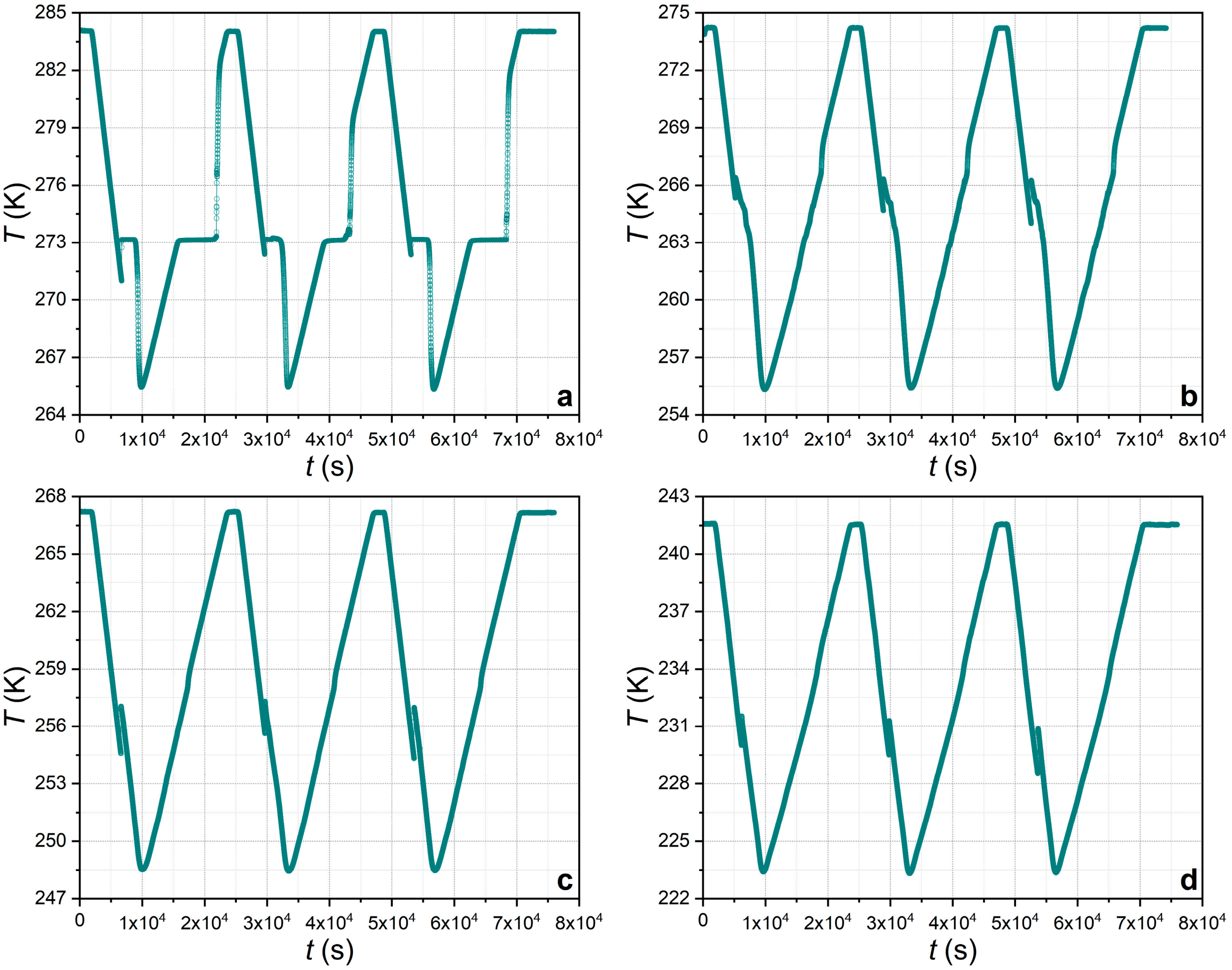

Figure S3. **(a)** Thermal curves of three cycles of cooling (10 K/h) – heating (5 K/h), obtained by measuring the ice crystallization and melting temperatures for pure water; **(b)** the same for 10.03 mass% aqueous MeOH solution; **(c)** the same for 20 mass% aqueous MeOH solution; **(d)** the same for 40 mass% aqueous MeOH solution; one temperature reading every 5 s.

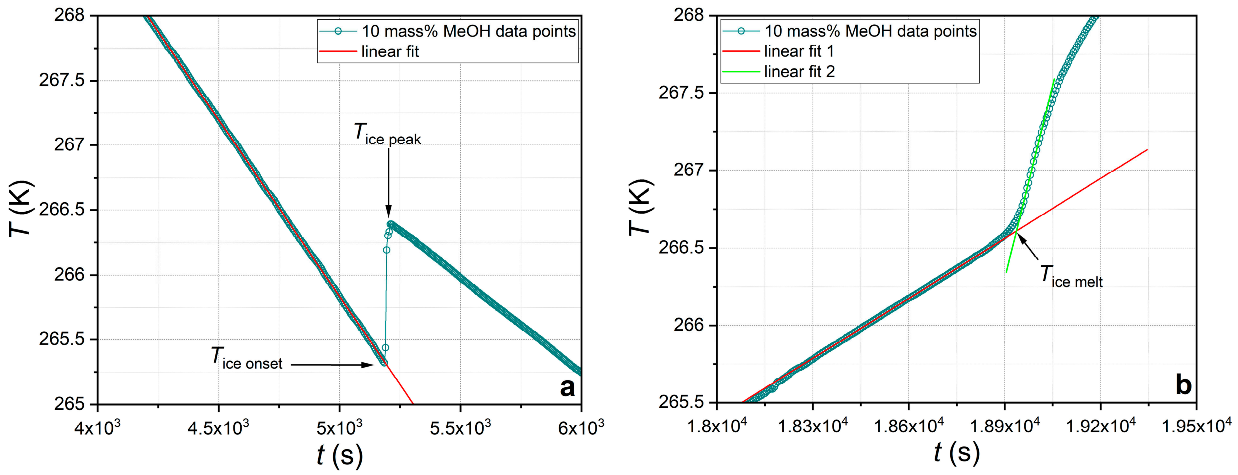

Figure S4. **(a)** Example of determining $T_{\text{ice onset}}$ and $T_{\text{ice peak}}$ temperatures during linear cooling at 10 K/h using a 10 mass% aqueous MeOH solution; **(b)** example of determining the ice melting temperature $T_{\text{ice melt}}$ during linear heating 5 K/h for the same sample; symbols – measured values (one point per 5 s), red and green lines – linear approximations; one temperature reading every 5 s.

Table S6. Experimental data on ice freezing and melting temperatures for aqueous $MgCl_2$ solutions; mean ± standard deviation values for three replicates are shown in bold.

$MgCl_2$ concentration, mass% (mol%)	$T_{ice\ onset}$, K	$T_{ice\ peak}$, K	$T_{ice\ melt}$, K
0 (0)	271.01	273.16	273.16
	272.38	273.16	273.16
	272.36	273.16	273.15
	**271.92±0.79**	**273.16±0.00**	**273.16±0.01**
5.13 (1.01)	266.68	269.95	270.12
	269.96	270.05	270.17
	269.13	270.06	270.16
	**263.51±0.48**	**269.82±0.03**	**270.15±0.04**
8.43 (1.71)	260.33	266.55	267.31
	260.39	266.56	267.24
	259.19	266.40	267.31
	**259.97±0.68**	**266.50±0.09**	**267.29±0.04**
12.63 (2.66)	253.59	260.25	262.09
	255.14	260.64	262.10
	255.20	260.68	262.11
	**254.64±0.91**	**260.52±0.24**	**262.10±0.01**
16.80 (3.68)	250.71	252.84	254.22
	253.31	253.68	254.25
	253.26	253.70	254.29
	**252.43±1.49**	**253.41±0.49**	**254.25±0.04**
19.97 (4.51)	244.25	244.64	245.54
	244.25	244.67	245.56
	244.32	244.71	245.56
	**244.27±0.04**	**244.67±0.03**	**245.55±0.01**
22.37 (5.17)	235.53 [a]	235.75 [a]	236.99 [a]
	235.61 [a]	235.82 [a]	237.09 [a]
	235.69 [a]	235.91 [a]	237.16 [a]
	**235.61±0.08** [a]	**235.83±0.08** [a]	**237.08±0.09** [a]
24.06 (5.66)	228.75 [a]	228.56 [a]	-
	228.93 [a]	228.75 [a]	229.97 [a]
	229.08 [a]	228.86 [a]	230.08 [a]
	**228.92±0.17** [a]	**228.73±0.15** [a]	**230.02±0.08** [a]

[a] The crystallization and melting temperatures of the metastable ice phase were measured for aqueous solutions of 22.37 and 24.06 mass% $MgCl_2$. For the $MgCl_2$–H_2O system at 0.1 MPa, the eutectic composition contains 21.6 mass% $MgCl_2$ and melts at 239.95 K (non-variant equilibrium of an aqueous salt solution, ice, and $MgCl_2 \cdot 12H_2O$ crystalline hydrate solid) [2]. Therefore, the thermodynamically stable solid phase for 22.37 and 24.06 mass% aqueous $MgCl_2$ solutions is $MgCl_2 \cdot 12H_2O$, which crystallizes at higher supercooling and melts at higher temperatures than metastable ice.

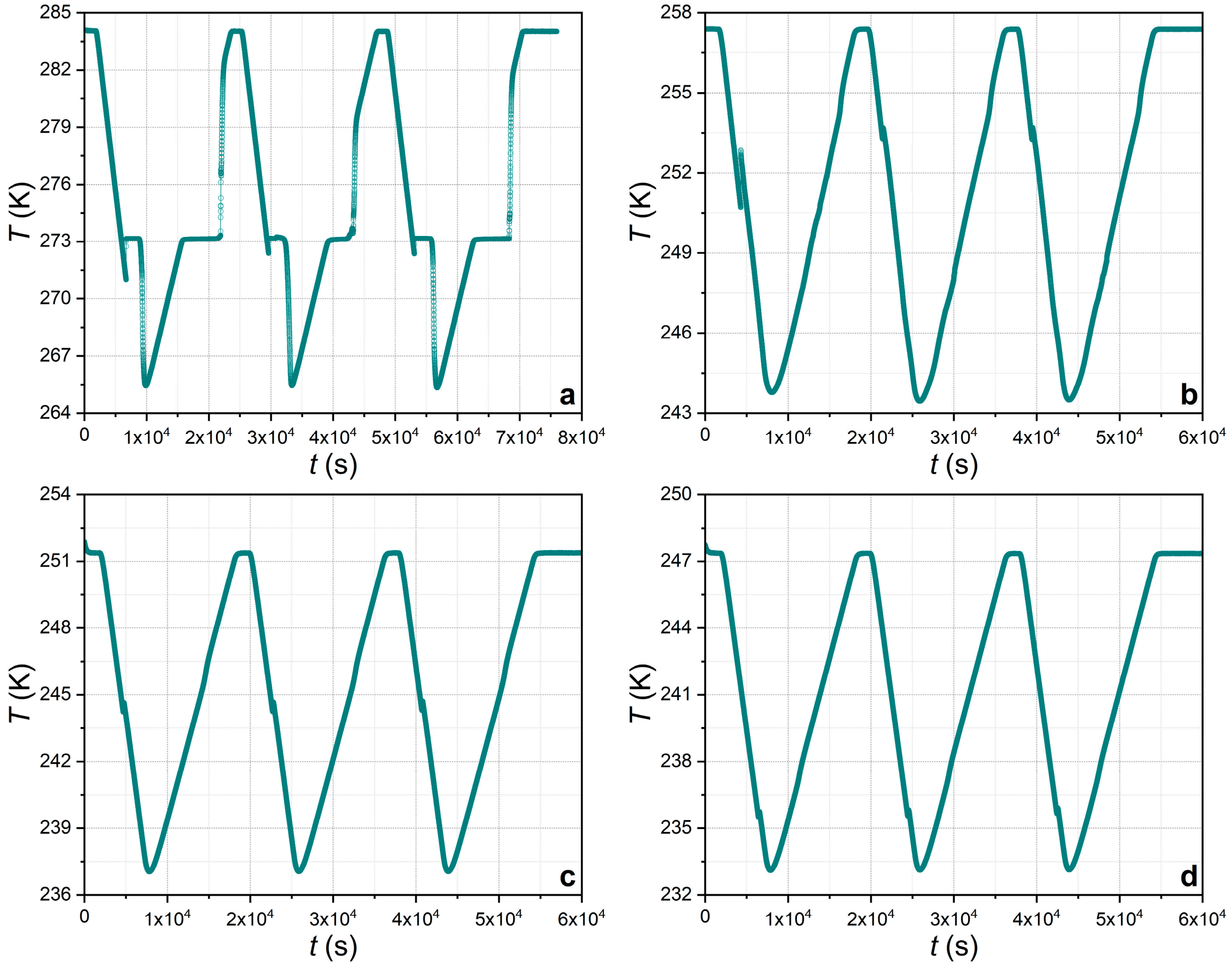

Figure S5. **(a)** Thermal curves of three cycles of cooling (10 K/h) – heating (5 K/h), obtained by measuring the ice crystallization and melting temperatures for pure water; **(b)** the same for 16.80 mass% aqueous $MgCl_2$ solution; **(c)** the same for 19.97 mass% aqueous $MgCl_2$ solution; **(d)** the same for 22.37 mass% aqueous $MgCl_2$ solution; one temperature reading every 5 s.

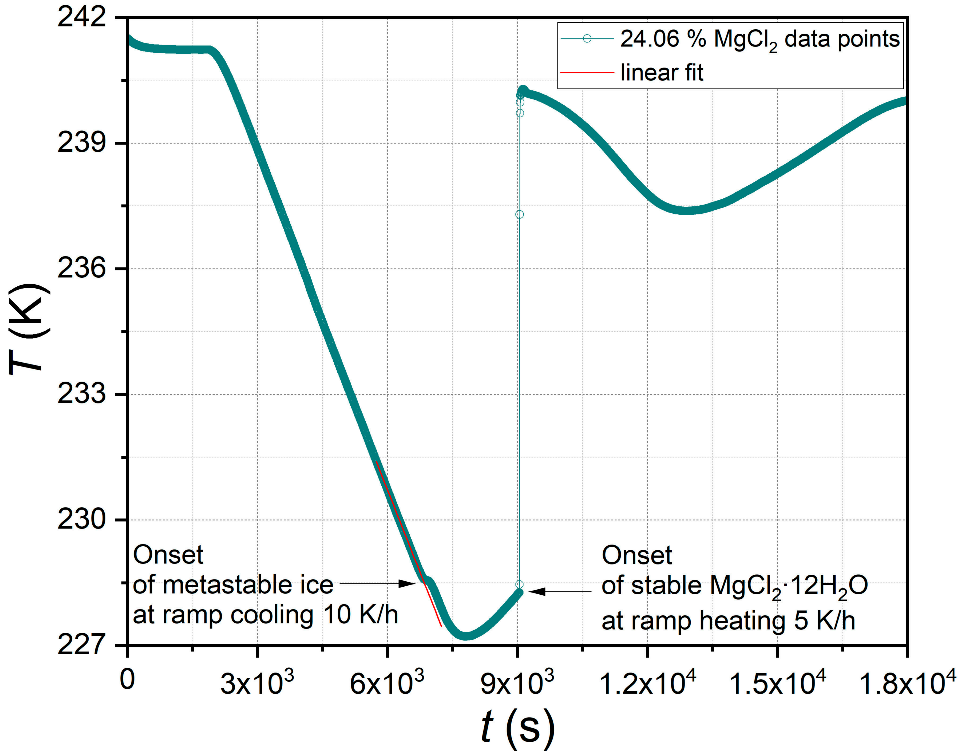

Figure S6. Thermal curves showing the sequential crystallization of the metastable ice phase and the thermodynamically stable $MgCl_2 \cdot 12H_2O$ phase for an aqueous solution containing 24.06 mass% $MgCl_2$; one temperature reading every 5 s.

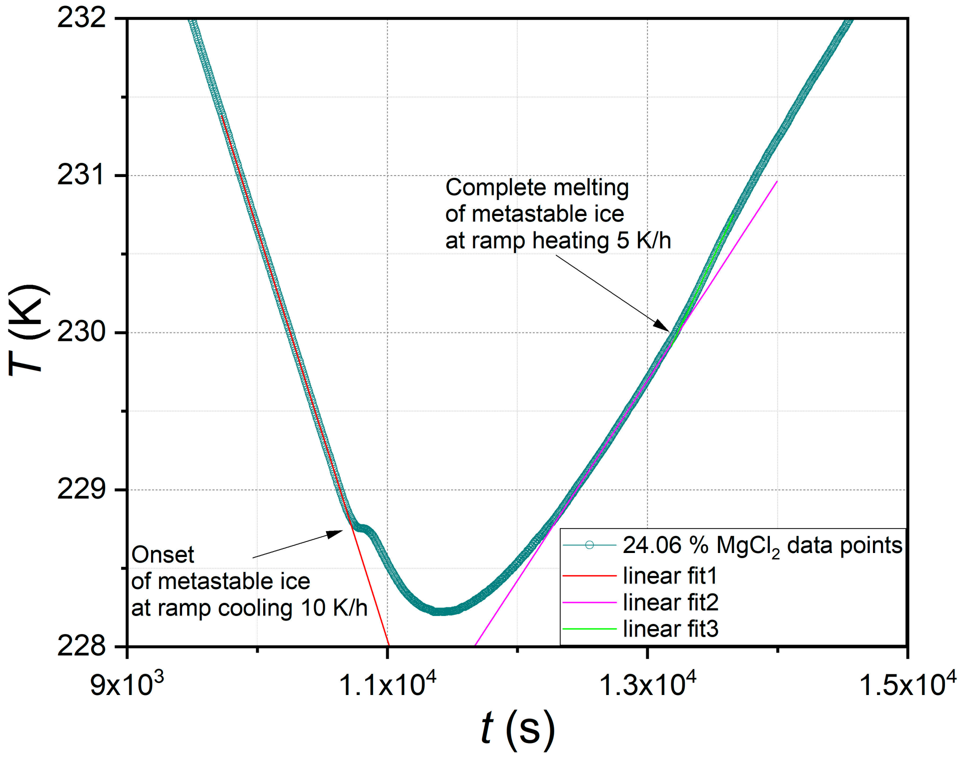

Figure S7. Thermal curves showing the crystallization and melting of the metastable ice phase for an aqueous solution containing 24.06 mass% $MgCl_2$; one temperature reading every 5 s.

References

[1] A. Semenov, R. Mendgaziev, A. Stoporev, V. Istomin, T. Tulegenov, M. Yarakhmedov, A. Novikov, V. Vinokurov, Direct Measurement of the Four-Phase Equilibrium Coexistence Vapor–Aqueous Solution–Ice–Gas Hydrate in Water–Carbon Dioxide System, Int. J. Mol. Sci. 24 (2023) 9321. https://doi.org/10.3390/ijms24119321.

[2] M. Tang, W.H. Tao, W.T. Huang, C.C. Huang, Y.P. Chen, Measurements of the Heat Capacity and Solid-liquid Equilibrium of Water-Potassium Chloride and Water-Magnesium Chloride Binary Mixtures, J. Chinese Inst. Chem. Eng. 33 (2002) 469–475. https://doi.org/10.6967/JCICE.200209.0469.
